# 1-[(*E*)-(3,4-Dimethyl­isoxazol-5-yl)imino­meth­yl]-2-naphthol

**DOI:** 10.1107/S160053681001216X

**Published:** 2010-04-10

**Authors:** Hoong-Kun Fun, Madhukar Hemamalini, Abdullah M. Asiri, Salman A. Khan

**Affiliations:** a X-ray Crystallography Unit, School of Physics, Universiti Sains Malaysia, 11800 USM, Penang, Malaysia; b Department of Chemistry, Faculty of Science, King Abdu Aziz University, Jeddah, Saudi Arabia

## Abstract

The title Schiff base compound, C_16_H_14_N_2_O_2_, has been synthesized by the reaction of 5-amino-3,4-dimethyl­isoxazole and 2-hydr­oxy-1-naphthaldehyde. The dihedral angle between the isoxazole ring and the napthyl ring system is 3.29 (7)°. The mol­ecule adopts an *E* configuration about the central C=N double bond. Intra­molecular O—H⋯N hydrogen bonding generates an *S*(6) ring motif. In the crystal structure, π–π inter­actions are observed involving the isoxazole ring and the substituted benzene ring of the naphthyl unit, with centroid–centroid distances of 3.5200 (10) Å.

## Related literature

For related background and the biological activity of isoxazol, see: Howell & Kimmel (2008 ▶); Bartlett & Schleyerbach (1985 ▶); Lamani *et al.* (2009 ▶); Jayashankar *et al.* (2009 ▶). For related structures, see: Alvarez-Thon *et al.* (2006 ▶); Tahir *et al.* (2008 ▶); Shad *et al.* (2008 ▶); Fun *et al.* (2010 ▶). For details of hydrogen-bond motifs, see: Bernstein *et al.* (1995 ▶). For the stability of the temperature controller used in the data collection, see: Cosier & Glazer (1986 ▶).
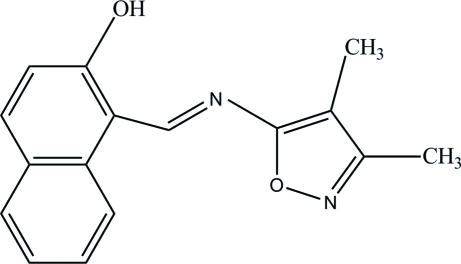


## Experimental

### 

#### Crystal data

C_16_H_14_N_2_O_2_
*M* *_r_* = 266.29
Monoclinic, 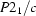

*a* = 7.5250 (6) Å
*b* = 15.4643 (12) Å
*c* = 12.3982 (7) Å
β = 117.377 (4)°
*V* = 1281.17 (16) Å^3^
*Z* = 4
Mo *K*α radiation
μ = 0.09 mm^−1^
*T* = 100 K
0.79 × 0.06 × 0.05 mm

#### Data collection

Bruker APEX DUO CCD area-detector diffractometer
Absorption correction: multi-scan (*SADABS*; Bruker, 2009 ▶) *T*_min_ = 0.930, *T*_max_ = 0.996
16577 measured reflections
3704 independent reflections
2843 reflections with *I* > 2σ(*I*)
*R*_int_ = 0.041

#### Refinement

*R*[*F* ^2^ > 2σ(*F* ^2^)] = 0.046
*wR*(*F* ^2^) = 0.134
*S* = 1.05
3704 reflections
237 parameters
H atoms treated by a mixture of independent and constrained refinement
Δρ_max_ = 0.45 e Å^−3^
Δρ_min_ = −0.23 e Å^−3^

### 

Data collection: *APEX2* (Bruker, 2009 ▶); cell refinement: *SAINT* (Bruker, 2009 ▶); data reduction: *SAINT*; program(s) used to solve structure: *SHELXTL* (Sheldrick, 2008 ▶); program(s) used to refine structure: *SHELXTL*; molecular graphics: *PLATON* (Spek, 2009 ▶); software used to prepare material for publication: *SHELXTL* and *PLATON*.

## Supplementary Material

Crystal structure: contains datablocks global, I. DOI: 10.1107/S160053681001216X/sj2761sup1.cif

Structure factors: contains datablocks I. DOI: 10.1107/S160053681001216X/sj2761Isup2.hkl

Additional supplementary materials:  crystallographic information; 3D view; checkCIF report

## Figures and Tables

**Table 1:** Hydrogen-bond geometry (Å, °)

*D*—H⋯*A*	*D*—H	H⋯*A*	*D*⋯*A*	*D*—H⋯*A*
O1—H1*O*1⋯N1	0.97 (2)	1.66 (3)	2.5471 (15)	150 (2)

**Fig. 1.**
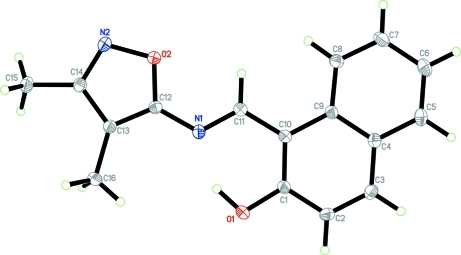
The asymmetric unit of the title compound, showing 50% probability displacement ellipsoids and the atom-numbering scheme.

**Fig. 2.**
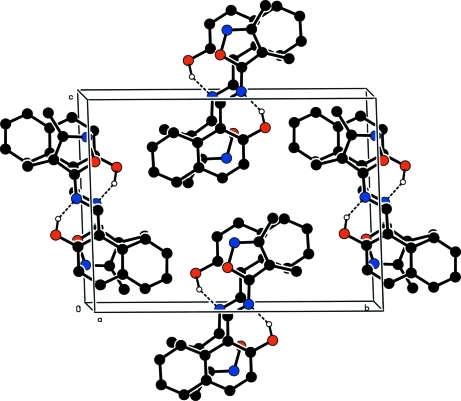
The crystal packing of the title compound, showing the hydrogen-bonded network (dashed lines). H atoms are not involved in hydrogen bond interactions are omitted for clarity.

## supplementary crystallographic information

### Comment

Five-membered heterocyclic compounds, natural as well as synthetic, are important for their biological activities. Compounds with isoxazol rings are of interest due to their broad spectrum of biological activities against monoamine oxidase inhibitor (Howell & Kimmel, 2008), bacterial (Bartlett & Schleyerbach, 1985), depression (Lamani *et al.*, 2009), hypertensive (Howell & Kimmel, 2008), pyretic and inflammatory diseases (Jayashankar *et al.*, 2009). The crystal structures of 2-[(*E*)-(3,5-dimethylisoxazol- 4-yl)diazenyl]benzoic acid (Alvarez-Thon *et al.*, 2006), 4-Bromo-2-((*E*)-{4-[(3,4-dimethylisoxazol-5-yl)sulfamoyl]phenyl}iminiomethyl)phenolate (Tahir *et al.*, 2008), 4-Chloro-2-[(*E*)-({4-[N-(3,4-dimethyl isoxazol-5-yl)sulfamoyl]phenyl}iminio)methyl]phenolate (Shad *et al.*, 2008) and 2-[(*E*)-(3,4-Dimethylisoxazol-5-yl) iminomethyl]phenol (Fun *et al.*, 2010) have been reported previously. In view of the importance of the title compound, (I), its crystal structure is reported here.

In the title compound (Fig. 1), the isoxazole ring is essentially planar with a maximum deviation of  0.007 (2) Å for atom C13. The dihedral angle between the isoxazole (O2/N2/C12–C14) ring and the (C1–C4/C9–C10) ring of the naphthyl unit, is 3.29 (7)°. The C12—O2 and C11═N1 bond lengths are 1.3635 (14) Å and 1.3036 (15) Å, respectively, and agree with the corresponding values in 2-[(*E*)-(3,4-dimethylisoxazol-5-yl)iminomethyl]phenol [1.344 (3) and 1.292 (4) Å; Fun *et al.*, 2010].

In the crystal structure (Fig. 2), the imino N atoms are linked to the phenol O atoms and act as hydrogen-bond acceptors in intramolecular O1—H1O1···N1 interactions (Table 1) , which generate *S*(6) ring motifs (Bernstein *et al.*, 1995). The crystal structure is further stabilized by π–π interactions involving the isoxazole (O2/N2/C12–C14)ring and the (C1–C4/C9–C10) ring of the naphthyl unit, with centroid to centroid distance of 3.5200 (10) Å [symmetry code: -x, 2-y, 1-z].

### Experimental

A mixture of 5-amino-3,4-dimethylisoxazole (0.50 g, 0.0025 mol) and 2-hydroxy-1-naphthaledhyde (0.43 g, 0.0025 mol) in methanol (15 mL) was refluxed for 5 h with stirring to give a light yellow precipitate. Then it was filtered and washed with methanol to give the pure compound. Yield: 72%; m. p. 160° C. The sample was recrystalized from methanol by dissolving the crude product and leaving the solution to evaporate slowly at room temperature. IR (KBr) v(max) cm^-1^: 2933 (C—H aromatic), 1626 (C═C), 1585 (HC═N), 1123 (C—N). ^1^H NMR (600 MHz, CDCl_3_) d: 8.30 (H3, d, J=12.72 Hz), 7.99 (H4, d, J=13.5 Hz), 7.87 (H5, d, J=11.76 Hz), 7.70 (H6, dd, J=8.58 Hz, J=5.1 Hz), 7.49 (H7, dd, J=10.8 Hz, J= 5.4 Hz), 7.34 (H9, s), 2.36 (-CH3, s), 2.15 (-CH3, s).

### Refinement

All the H atoms were located in a difference Fourier map and allowed to refine freely [O—H = 0.97 (2) Å, C—H = 0.916 (19)–1.004 (19) Å].

### Crystal data

**Table:**

C_16_H_14_N_2_O_2_	*F*(000) = 560
*M**_r_* = 266.29	*D*_x_ = 1.381 Mg m^−^^3^
Monoclinic, *P*2_1_/*c*	Mo *K*α radiation, λ = 0.71073 Å
Hall symbol: -P 2ybc	Cell parameters from 3616 reflections
*a* = 7.5250 (6) Å	θ = 2.6–34.2°
*b* = 15.4643 (12) Å	µ = 0.09 mm^−^^1^
*c* = 12.3982 (7) Å	*T* = 100 K
β = 117.377 (4)°	Needle, yellow
*V* = 1281.17 (16) Å^3^	0.79 × 0.06 × 0.05 mm
*Z* = 4	

### Data collection

**Table:**

Bruker APEX DUO CCD area-detector diffractometer	3704 independent reflections
Radiation source: fine-focus sealed tube	2843 reflections with *I* > 2σ(*I*)
graphite	*R*_int_ = 0.041
φ and ω scans	θ_max_ = 30.0°, θ_min_ = 2.3°
Absorption correction: multi-scan (*SADABS*; Bruker, 2009)	*h* = −10→10
*T*_min_ = 0.930, *T*_max_ = 0.996	*k* = −21→21
16577 measured reflections	*l* = −17→17

### Refinement

**Table:**

Refinement on *F*^2^	Primary atom site location: structure-invariant direct methods
Least-squares matrix: full	Secondary atom site location: difference Fourier map
*R*[*F*^2^ > 2σ(*F*^2^)] = 0.046	Hydrogen site location: inferred from neighbouring sites
*wR*(*F*^2^) = 0.134	H atoms treated by a mixture of independent and constrained refinement
*S* = 1.05	*w* = 1/[σ^2^(*F*_o_^2^) + (0.0698*P*)^2^ + 0.3682*P*] where *P* = (*F*_o_^2^ + 2*F*_c_^2^)/3
3704 reflections	(Δ/σ)_max_ = 0.001
237 parameters	Δρ_max_ = 0.45 e Å^−^^3^
0 restraints	Δρ_min_ = −0.23 e Å^−^^3^

### Special details

**Table:**

Experimental. The crystal was placed in the cold stream of an Oxford Cryosystems Cobra open-flow nitrogen cryostat (Cosier & Glazer, 1986) operating at 100.0 (1) K.
Geometry. All s.u.'s (except the s.u. in the dihedral angle between two l.s. planes) are estimated using the full covariance matrix. The cell s.u.'s are taken into account individually in the estimation of s.u.'s in distances, angles and torsion angles; correlations between s.u.'s in cell parameters are only used when they are defined by crystal symmetry. An approximate (isotropic) treatment of cell s.u.'s is used for estimating s.u.'s involving l.s. planes.
Refinement. Refinement of F^2^ against ALL reflections. The weighted R-factor wR and goodness of fit S are based on F^2^, conventional R-factors R are based on F, with F set to zero for negative F^2^. The threshold expression of F^2^ > 2σ(F^2^) is used only for calculating R-factors(gt) etc. and is not relevant to the choice of reflections for refinement. R-factors based on F^2^ are statistically about twice as large as those based on F, and R- factors based on ALL data will be even larger.

### Fractional atomic coordinates and isotropic or equivalent isotropic displacement parameters (Å^2^)

**Table:**

	*x*	*y*	*z*	*U*_iso_*/*U*_eq_	
O1	0.20289 (17)	1.11942 (6)	0.64493 (8)	0.0209 (2)	
O2	0.24094 (15)	0.97368 (6)	0.30327 (8)	0.0173 (2)	
N1	0.24292 (17)	1.04190 (6)	0.47613 (9)	0.0152 (2)	
N2	0.21030 (18)	0.99921 (7)	0.18571 (9)	0.0182 (2)	
C1	0.2410 (2)	1.04273 (7)	0.70239 (11)	0.0150 (2)	
C2	0.2479 (2)	1.04255 (8)	0.81837 (11)	0.0179 (3)	
C3	0.2877 (2)	0.96778 (8)	0.88407 (11)	0.0172 (3)	
C4	0.3177 (2)	0.88841 (8)	0.83642 (11)	0.0149 (2)	
C5	0.3597 (2)	0.81113 (8)	0.90574 (12)	0.0184 (3)	
C6	0.3833 (2)	0.73399 (8)	0.85954 (12)	0.0201 (3)	
C7	0.3626 (2)	0.73093 (8)	0.74058 (12)	0.0207 (3)	
C8	0.3253 (2)	0.80526 (8)	0.67215 (12)	0.0177 (3)	
C9	0.30404 (19)	0.88660 (7)	0.71809 (11)	0.0141 (2)	
C10	0.26908 (19)	0.96648 (7)	0.65075 (11)	0.0138 (2)	
C11	0.2611 (2)	0.96861 (8)	0.53223 (11)	0.0148 (2)	
C12	0.2289 (2)	1.04637 (7)	0.36180 (11)	0.0146 (2)	
C13	0.1957 (2)	1.11763 (7)	0.29069 (11)	0.0140 (2)	
C14	0.18303 (19)	1.08332 (8)	0.18048 (11)	0.0151 (2)	
C15	0.1393 (2)	1.13268 (9)	0.06759 (12)	0.0201 (3)	
C16	0.1737 (2)	1.20938 (8)	0.31960 (12)	0.0175 (3)	
H2A	0.222 (3)	1.0979 (12)	0.8512 (16)	0.029 (5)*	
H3A	0.290 (3)	0.9681 (11)	0.9648 (16)	0.024 (4)*	
H5A	0.375 (3)	0.8132 (11)	0.9894 (16)	0.027 (4)*	
H6A	0.416 (3)	0.6822 (12)	0.9078 (16)	0.027 (4)*	
H7A	0.376 (3)	0.6775 (12)	0.7059 (16)	0.029 (5)*	
H8A	0.309 (3)	0.8009 (11)	0.5908 (15)	0.022 (4)*	
H11A	0.272 (3)	0.9140 (11)	0.4942 (15)	0.026 (4)*	
H15A	0.091 (3)	1.0970 (12)	0.0011 (17)	0.032 (5)*	
H15B	0.256 (3)	1.1642 (14)	0.0772 (18)	0.043 (6)*	
H15C	0.034 (3)	1.1763 (14)	0.0517 (18)	0.043 (6)*	
H16A	0.202 (3)	1.2197 (12)	0.4021 (18)	0.036 (5)*	
H16B	0.263 (4)	1.2459 (16)	0.302 (2)	0.053 (6)*	
H16C	0.041 (4)	1.2329 (14)	0.266 (2)	0.047 (6)*	
H1O1	0.207 (4)	1.1091 (15)	0.569 (2)	0.055 (7)*	

### Atomic displacement parameters (Å^2^)

**Table:**

	*U*^11^	*U*^22^	*U*^33^	*U*^12^	*U*^13^	*U*^23^
O1	0.0339 (6)	0.0115 (4)	0.0197 (4)	0.0004 (4)	0.0144 (4)	0.0013 (3)
O2	0.0244 (5)	0.0130 (4)	0.0163 (4)	0.0015 (4)	0.0109 (4)	0.0002 (3)
N1	0.0175 (5)	0.0146 (5)	0.0133 (5)	0.0000 (4)	0.0069 (4)	0.0012 (3)
N2	0.0232 (6)	0.0186 (5)	0.0150 (5)	0.0001 (4)	0.0107 (5)	0.0001 (4)
C1	0.0173 (6)	0.0123 (5)	0.0150 (5)	−0.0015 (4)	0.0070 (5)	0.0004 (4)
C2	0.0223 (7)	0.0155 (5)	0.0166 (5)	−0.0020 (5)	0.0095 (5)	−0.0032 (4)
C3	0.0189 (6)	0.0188 (6)	0.0143 (5)	−0.0033 (5)	0.0080 (5)	−0.0013 (4)
C4	0.0135 (6)	0.0160 (5)	0.0149 (5)	−0.0009 (4)	0.0061 (5)	0.0014 (4)
C5	0.0183 (6)	0.0204 (6)	0.0170 (5)	−0.0006 (5)	0.0085 (5)	0.0046 (4)
C6	0.0193 (6)	0.0171 (6)	0.0246 (6)	0.0001 (5)	0.0106 (5)	0.0063 (5)
C7	0.0240 (7)	0.0147 (6)	0.0261 (6)	0.0033 (5)	0.0139 (6)	0.0029 (5)
C8	0.0218 (7)	0.0146 (5)	0.0199 (6)	0.0017 (5)	0.0123 (5)	0.0007 (4)
C9	0.0141 (6)	0.0137 (5)	0.0150 (5)	0.0004 (4)	0.0072 (5)	0.0013 (4)
C10	0.0148 (6)	0.0125 (5)	0.0144 (5)	0.0000 (4)	0.0068 (5)	0.0003 (4)
C11	0.0151 (6)	0.0139 (5)	0.0153 (5)	0.0004 (5)	0.0069 (5)	0.0004 (4)
C12	0.0158 (6)	0.0141 (5)	0.0132 (5)	−0.0011 (5)	0.0062 (5)	−0.0014 (4)
C13	0.0147 (6)	0.0136 (5)	0.0139 (5)	−0.0009 (4)	0.0066 (5)	−0.0003 (4)
C14	0.0142 (6)	0.0166 (5)	0.0152 (5)	−0.0005 (5)	0.0073 (5)	0.0001 (4)
C15	0.0244 (7)	0.0220 (6)	0.0153 (6)	−0.0011 (6)	0.0105 (5)	0.0017 (5)
C16	0.0216 (7)	0.0130 (5)	0.0175 (6)	0.0006 (5)	0.0085 (5)	0.0001 (4)

### Geometric parameters (Å, °)

**Table:**

O1—C1	1.3448 (14)	C6—H6A	0.962 (18)
O1—H1O1	0.97 (2)	C7—C8	1.3787 (17)
O2—C12	1.3635 (14)	C7—H7A	0.957 (19)
O2—N2	1.4229 (13)	C8—C9	1.4198 (16)
N1—C11	1.3036 (15)	C8—H8A	0.962 (17)
N1—C12	1.3739 (15)	C9—C10	1.4457 (16)
N2—C14	1.3138 (16)	C10—C11	1.4431 (16)
C1—C10	1.4033 (16)	C11—H11A	0.989 (18)
C1—C2	1.4147 (17)	C12—C13	1.3607 (16)
C2—C3	1.3659 (17)	C13—C14	1.4274 (16)
C2—H2A	1.004 (19)	C13—C16	1.4912 (16)
C3—C4	1.4245 (17)	C14—C15	1.4920 (17)
C3—H3A	0.991 (18)	C15—H15A	0.916 (19)
C4—C5	1.4203 (17)	C15—H15B	0.96 (2)
C4—C9	1.4231 (16)	C15—H15C	0.99 (2)
C5—C6	1.3696 (19)	C16—H16A	0.96 (2)
C5—H5A	0.990 (18)	C16—H16B	0.98 (3)
C6—C7	1.4108 (19)	C16—H16C	0.98 (2)
			
C1—O1—H1O1	105.9 (14)	C8—C9—C10	123.34 (11)
C12—O2—N2	107.29 (9)	C4—C9—C10	119.09 (11)
C11—N1—C12	122.26 (10)	C1—C10—C11	120.06 (10)
C14—N2—O2	105.86 (9)	C1—C10—C9	118.69 (11)
O1—C1—C10	122.65 (11)	C11—C10—C9	121.25 (10)
O1—C1—C2	116.06 (10)	N1—C11—C10	120.54 (11)
C10—C1—C2	121.28 (11)	N1—C11—H11A	120.0 (10)
C3—C2—C1	120.22 (11)	C10—C11—H11A	119.5 (10)
C3—C2—H2A	120.6 (10)	C13—C12—O2	111.11 (10)
C1—C2—H2A	119.2 (10)	C13—C12—N1	127.84 (11)
C2—C3—C4	121.00 (11)	O2—C12—N1	121.01 (10)
C2—C3—H3A	119.7 (10)	C12—C13—C14	103.35 (10)
C4—C3—H3A	119.3 (10)	C12—C13—C16	128.48 (11)
C5—C4—C9	119.85 (11)	C14—C13—C16	128.16 (11)
C5—C4—C3	120.52 (11)	N2—C14—C13	112.37 (11)
C9—C4—C3	119.63 (11)	N2—C14—C15	120.98 (11)
C6—C5—C4	121.03 (12)	C13—C14—C15	126.63 (11)
C6—C5—H5A	119.4 (10)	C14—C15—H15A	111.2 (12)
C4—C5—H5A	119.5 (10)	C14—C15—H15B	110.3 (12)
C5—C6—C7	119.55 (12)	H15A—C15—H15B	112.1 (18)
C5—C6—H6A	120.8 (11)	C14—C15—H15C	110.3 (12)
C7—C6—H6A	119.7 (11)	H15A—C15—H15C	106.4 (16)
C8—C7—C6	120.58 (12)	H15B—C15—H15C	106.3 (18)
C8—C7—H7A	118.5 (11)	C13—C16—H16A	114.9 (11)
C6—C7—H7A	120.9 (11)	C13—C16—H16B	109.5 (14)
C7—C8—C9	121.36 (12)	H16A—C16—H16B	107.7 (18)
C7—C8—H8A	118.6 (10)	C13—C16—H16C	112.3 (13)
C9—C8—H8A	120.0 (10)	H16A—C16—H16C	108.8 (19)
C8—C9—C4	117.57 (11)	H16B—C16—H16C	102.8 (19)
			
C12—O2—N2—C14	0.20 (14)	C8—C9—C10—C1	177.05 (13)
O1—C1—C2—C3	−179.34 (12)	C4—C9—C10—C1	−2.75 (19)
C10—C1—C2—C3	1.7 (2)	C8—C9—C10—C11	−2.4 (2)
C1—C2—C3—C4	−1.7 (2)	C4—C9—C10—C11	177.78 (12)
C2—C3—C4—C5	179.74 (13)	C12—N1—C11—C10	−178.04 (12)
C2—C3—C4—C9	−0.7 (2)	C1—C10—C11—N1	5.2 (2)
C9—C4—C5—C6	−1.5 (2)	C9—C10—C11—N1	−175.36 (12)
C3—C4—C5—C6	178.15 (13)	N2—O2—C12—C13	−1.01 (15)
C4—C5—C6—C7	−0.9 (2)	N2—O2—C12—N1	176.88 (12)
C5—C6—C7—C8	2.1 (2)	C11—N1—C12—C13	175.02 (14)
C6—C7—C8—C9	−0.8 (2)	C11—N1—C12—O2	−2.5 (2)
C7—C8—C9—C4	−1.5 (2)	O2—C12—C13—C14	1.33 (15)
C7—C8—C9—C10	178.66 (13)	N1—C12—C13—C14	−176.38 (13)
C5—C4—C9—C8	2.64 (19)	O2—C12—C13—C16	−179.77 (13)
C3—C4—C9—C8	−176.96 (12)	N1—C12—C13—C16	2.5 (2)
C5—C4—C9—C10	−177.54 (12)	O2—N2—C14—C13	0.64 (15)
C3—C4—C9—C10	2.85 (19)	O2—N2—C14—C15	−177.96 (12)
O1—C1—C10—C11	1.1 (2)	C12—C13—C14—N2	−1.23 (16)
C2—C1—C10—C11	179.96 (13)	C16—C13—C14—N2	179.86 (13)
O1—C1—C10—C9	−178.36 (12)	C12—C13—C14—C15	177.28 (13)
C2—C1—C10—C9	0.5 (2)	C16—C13—C14—C15	−1.6 (2)

### Hydrogen-bond geometry (Å, °)

**Table:**

*D*—H···*A*	*D*—H	H···*A*	*D*···*A*	*D*—H···*A*
O1—H1O1···N1	0.97 (2)	1.66 (3)	2.5471 (15)	150 (2)
